# Novel Approach to Identify Potential Bioactive Plant Metabolites: Pharmacological and Metabolomics Analyses of Ethanol and Hot Water Extracts of Several Canadian Medicinal Plants of the Cree of Eeyou Istchee

**DOI:** 10.1371/journal.pone.0135721

**Published:** 2015-08-11

**Authors:** Nan Shang, Ammar Saleem, Lina Musallam, Brendan Walshe-Roussel, Alaa Badawi, Alain Cuerrier, John T. Arnason, Pierre S. Haddad

**Affiliations:** 1 Natural Health Products and Metabolic Disease Laboratory and Montreal Diabetes Research Center, Department of pharmacology, Université de Montréal, Montreal, Quebec, Canada; 2 Canadian Institutes of Health Research Team in Aboriginal Antidiabetic Medicines, Montreal, Quebec, Canada; 3 Institute of Nutraceuticals and Functional Foods, Université Laval, Quebec, Quebec, Canada; 4 Center for Advanced Research in Environmental Genomics, University of Ottawa, Ottawa, Ontario, Canada; 5 Office for Biotechnology, Genomics and Population Health, Public Health Agency of Canada, Toronto, Ontario, Canada; 6 Jardin botanique de Montréal, Institut de recherche en biologie végétale, Université de Montréal, Montréal, Quebec, Canada; USDA-ARS, UNITED STATES

## Abstract

We evaluated and compared the antidiabetic potential and molecular mechanisms of 17 Cree plants’ ethanol extracts (EE) and hot water extracts (HWE) on glucose homeostasis *in vitro* and used metabolomics to seek links with the content of specific phytochemicals. Several EE of medical plants stimulated muscle glucose uptake and inhibited hepatic G6Pase activity. Some HWE partially or completely lost these antidiabetic activities in comparison to EE. Only *R*. *groenlandicum* retained similar potential between EE and HWE in both assays. In C2C12 muscle cells, EE of *R*. *groenlandicum*, *A*. *incana* and *S*. *purpurea* stimulated glucose uptake by activating AMP-activated protein kinase (AMPK) pathway and increasing glucose transporter type 4 (GLUT4) expression. In comparison to EE, HWE of *R*. *groenlandicum* exhibited similar activities; HWE of *A*. *incana* completely lost its effect on all parameters; interestingly, HWE of *S*. *purpurea* activated insulin pathway instead of AMPK pathway to increase glucose uptake. In the liver, for a subset of 5 plants, HWE and EE activated AMPK pathway whereas the EE and HWE of *S*. *purpurea* and *K*. *angustifolia* also activated insulin pathways. Quercetin-3-O-galactoside and quercetin 3-O-α-L-arabinopyranoside, were successfully identified by discriminant analysis as biomarkers of HWE plant extracts that stimulate glucose uptake in vitro. More importantly, the latter compound was not identified by previous bioassay-guided fractionation.

## Introduction

Type 2 diabetes (T2D) is characterized by impaired insulin secretion and / or insulin sensitivity. In 2013, there were 346 million people worldwide having diabetes [1]. In particular, T2D is more pronounced among Indigenous populations, especially the Cree of Eeyou Istchee (CEI) of Canada, where the age-adjusted prevalence of T2D reached 29% in 2009 [2,3]. The burden of T2D and the ensuing deleterious complications (cardiovascular, retinopathy and nephropathy) prompted the search for culturally adapted treatment options for these Indigenous populations.

In order to identify such culturally adapted T2D complementary therapies, quantitative ethnobotanical studies have been performed by our team [2]. Seventeen plant extracts were tested for their antidiabetic potential in several screening studies through a variety of in vitro bioassays. Results showed that several extracts strongly stimulated glucose uptake (GU) in C2C12 cells and inhibited Glucose-6-Phosphatase (G6Pase) activity in H4IIE cells [4–7]. Indeed, the muscle is the main tissue regulating the postprandrial glucose, occurring principally through glucose transporter type 4 (GLUT4) [8]. On the other hand, G6Pase is the rate-limiting enzyme for the final step of gluconeogenesis and glycogenolysis, two pathways controlling hepatic glucose production (HGP) [9]. In T2D, unsuppressed HGP has been linked to increased G6Pase activity [10].

In these previous studies, a well-established and standard phytochemical extraction method was used, based on 80% aqueous ethanol to prepare crude plant extracts. However, traditional preparations of medicinal plants rely on other methods, such as hot water extraction. The first aim of the present study was therefore to evaluate and compare the biological activity of ethanol and hot water extracts (EE and HWE, respectively) of the 17 identified putative antidiabetic plant species from CEI traditional antidiabetic pharmacopeia. Secondly, we also began examining the molecular mechanisms underlying the modulating action of a subset of plants on glucose metabolism using the same in vitro bioassays. Finally, we applied plant metabolomics methods to obtain a detailed characterization of natural compounds present in all 17 species. Principal component analysis and discriminant analysis were then performed to identify potential links between phytochemical constituents in the various plant extracts and their biological activity. We report that this novel cross-disciplinary approach succeeds in identifying key biologically active constituents.

## Materials and Methods

### Plant material and extraction

The 17 Cree medicinal plant species used in our project, [2] as well as the concentrations used in different cell lines are listed in Table 1. Plant samples were collected for each species in two areas of the CEI territory and prepared as previously described [5,6]. Authorization for plant collection was ascertained through a comprehensive research agreement intervening between the participating Cree First Nations, the three Canadian universities (Université de Montréal, McGill University, University of Ottawa) and the Cree Board of Health and Social Services of James Bay. Dr. Alain Cuerrier, plant taxonomist at the Montreal Botanical Garden, ascertained the botanical identity of the plant species.

**Table 1:** List of investigated plant species and the concentrations of the extracts tested in cells.

Species	Abbreviation	Plant part	C2C12 (μg/mL)	H4II E (μg/mL)
*Rhododendron groenlandicum* (Oeder) Kron and Judd	*R*.*groenlandicum*	Leaves	75	50
*Abies balsamea* (L.) Mill.	*A*.*balsamea*	Bark	50	50
*Larix laricina* Du Roi (K.Koch)	*L*.*laricina*	Bark	25	25
*Picea mariana* (P. Mill.) BSP	*P*.*mariana*	Cones	10	10
*Sorbus decora* (Sarg.) C.K.Schneid.	*S*.*decora*	Bark	15	15
*Alnus incana* subsp. rugosa (Du Roi) R.T. Clausen	*A*.*incana*	Bark	50	50
*Sarracenia purpurea* L.	*S*.*purpurea*	Whole plant	100	25
*Pinus banksiana* Lamb.	*P*.*banksiana*	Cones	15	10
*Rhododendron tomentosum* (Stokes) Harmaja subsp.subarcticum (Harmaja) G.Wallace	*R*.*tomentosum*	Leaves	50	50
*Kalmia angustifolia* L.	*K*.*augustifolia*	Leaves	50	50
*Picea glauca* (Moench) Voss	*P*.*glauca*	Leaves	125	125
*Juniperus communis* L.	*J*.*communis*	Berries	5	3.75
*Salix planifolia* Pursh	*S*.*planifolia*	Bark	25	15
*Lycopodium clavatum* L.	*L*.*clavatum*	Whole plant	100	100
*Populus balsamifera* L.	*P*.*balsamifera*	Bark	100	100
*Gaultheria hispidula* (L.) Muhl.	*G*.*hispidula*	Leaves	25	25
*Vaccinium vitis-idaea* L.	*V*.*vitis-idaea*	Berries	200	200

Voucher specimens are deposited in the herbarium of the Montreal Botanical Garden in Montreal, Quebec, Canada. The collected plant samples were treated as previously described. Briefly, the plant material was extracted in two ways: the first (standard phytochemical extraction) method used 80% ethanol (10 mL/g dry material) and material was extracted twice for 24 h on a mechanical shaker (hereafter designated as EE); the second method (mimicking CEI traditional preparation) used boiling water for 75 min (hereafter designated as HWE). In both cases, extracts were vacuum filtered with Whatman Qualitative Grade 1 filter disc paper. EE were subsequently dried using a rotary evaporator at 40°C followed by lyophilization. HWE were dried using a spray dryer followed by lyophilization. All lyophilized extracts were preserved at 4°C in a desiccator. DMSO and warm water were used to reconstitute EE and HWE respectively. All the extracts were prepared as stock concentrations varying from 5–200 mg/ml and were added to the medium with the final concentrations shown in Table 1. The final concentration of DMSO was 0.1% for all the treatments.

### Phytochemical characterization of extracts and UPLC-QTOF analysis

Chromatography was carried out on Acquity BEH C18 column (1.7 μm 2.1 × 100 mm) connected with a VanGuard Pre-column 2.1 × 5 mm using an Acquity UPLC system with the column temperature at 50°C and sample temperature at 10°C. The mobile phase consisted of (A) water containing 0.1% formic acid and (B) acetonitrile containing 0.1% formic acid (Fisher Optima LC-MS). The gradient conditions of the mobile phase were: 0–1 min 5% B isocratic, 1–6 min linear gradient 5–50% B, 6–8 min 50–95%B, 8–10 min 5% B isocratic (total run time 10 min). The flow rate was 0.5 mL/min, and 1 μL of sample was injected followed by a strong wash 200 μL (90% acetonitrile+10% water) and weak wash 600 μL (10% acetonitrile+90% water).

Mass spectrometric analysis was performed using a Q-TOF mass spectrometer equipped with an electrospray ionization (ESI) interface (Xevo G2, Waters Inc.). The ESI source was operated in positive ionization mode with source temperature of 120°C, desolvation temperature of 400°C, Cone gas (N2) flow of 50 L/min, and desolvation gas (N2) flow of 1195 L/min. Leucine-enkephalin was used as the lock mass generating an [M+ H]+ ion (m/z 556. 2615). The optimal conditions used for MSe analysis were as follows: mass range 100–1500 Da, function 1 CE, 6V, function 2 CER 10–30V, cone voltage 20 V, scan time 0.1 sec. The system was calibrated with sodium formate and the data were acquired and processed with MassLynx (version 4.1) and MarkerLynx (version 8.03) software with principal component analysis (PCA). The retention times and the protonated masses were generated at a noise threshold of 500 counts and no smoothing was applied. Pareto scaling was applied to generate the score plots. To find out the discriminant biomarkers, the data were further analyzed by orthogonal partial least square discriminant analysis (OPLS-DA). S-plots were provided the relationship between covariance and correlation within the OPLS-DA results. The variables that contributed to discrimination between two groups were considered as potential biomarkers.

### Cell culture

The cell lines (C2C12 and H4IIE cell lines) were purchased from the American Type Culture Collection (ATCC), Manassas, USA. Other reagents were purchased from Invitrogen Life Technologies (Burlington, Canada) and Wisent (St. Bruno, Canada), Sigma-Aldrich (Oakville, Canada), unless otherwise specified below. All cells were cultured routinely in a incubator in a 5% CO_2_ at 37°C. The C2C12 myoblasts were grown in high-glucose Dulbecco’s modified Eagle medium (DMEM) supplemented with 10% fetal bovine serum (FBS), 10% horse serum (HS), and penicillin–streptomycin antibiotics. The 80% confluent C2C12 cells were differentiated for a period of 7 days into myotubes in differentiation medium (DMEM containing 2% HS and antibiotics). The H4IIE cells were grown in DMEM supplemented with 10% FBS and antibiotics and were ready for hepatic glucose production assay at 90% confluence.

### Glucose uptake bioassay

The ^3^H-deoxyglucose uptake assay was performed as described previously [5,6]. Briefly, on day 6 of differentiation, cells were treated for 18 hours in differentiation medium with either vehicle control (0.1% DMSO) alone, with a positive control Metformin (400 μM), extracts (EE and HWE respectively) re-suspended in vehicle at optimal concentrations, as shown in Table 1, or with pure compound of quercetin 3-O-α-L-arabinopyranoside (50 μM). Then, cells were rinsed twice with warm Krebs phosphate buffer, (KPB, 20 mM Hepes, 4.05 mM Na_2_HPO_4_, 0.95 mM NaH_2_PO_4_, 136 mM NaCl, 4.7 mM KCl, 1 mM CaCl_2_, and 1 mM MgSO_4_, 5 mM glucose, pH 7.4). A subset of vehicle wells was treated with 100 nM insulin in KPB for 30 min as a second positive control. Cells were then rinsed and incubated with 0.5 μCi/mL 2-deoxy- D-[1–3^H^]-glucose (TRK-383, Amersham Biosciences, Baie d’Urfé, Canada) for exactly 10 min. Cells were then rapidly rinsed and were lysed in 0.5 mL of 0.1 M NaOH for 30 min.The incorporated radioactivity of the lysates was measured in a scintillation beta counter (LKB Wallac 1219; Perkin-Elmer, Woodbridge, Ontario, Canada). Protein content was tested by the bicinchoninic acid (BCA) method (Thermo Scientific Pierce, Rockford, IL, USA) with bovine serum albumin (BSA) as standard protein.

### Hepatic Glucose Production and Glucose-6-phosphatase assay

The effects of plant extracts on HGP were assessed in H4IIE rat hepatoma cells with a Glucose-6-phosphatase (G6Pase) enzyme activity assay as described previously [7, 11]. Briefly, 90% confluent cells were treated for 18 hours with vehicle control, insulin (100 nM), or plants extracts (EE or HWE at optimal concentrations described in Table 1). Cells were then lysed in 15 mM phosphate buffer containing 0.05% Triton and 1.3 mM phenol (pH = 6.5). [7, 11] The lysates were incubated in the presence or absence of 200 mM glucose-6-phosphate (G6P) buffer for 40 min at 37°C. The glucose was produced using G6P as a substrate. Lysates of plant treated cells in the absence of G6P were used as negative controls. The glucose production was measured using Wako AutoKit Glucose (Wako Chemicals USA Inc., Richmond, VA, USA), according to the manufacturer's recommendations. Protein content was also quantified by the BCA method and the percent activity of vehicle control was shown as results.

### Western blot

Western blot was performed as previously described by our team [7,11]. Cells were lysed in RIPA lysis buffer (50 mM Hepes, 150 mM NaCl, 5 mM EGTA, 2 mM MgCl_2_.6H_2_O, 5% glycerol, 1% Trition X-100, 0.1% SDS, 1% Na-deoxycholate, 2mM phenylmethanesulfonyl fluoride (PMSF), 10 mM NaF, 100 μM Na-orthovanadate, 1 mM Na-pyrophosphate and 1 tablet of protease inhibitors (Complete Mini; Roche, Mannheim, Germany) at pH 7.4. A sucrose buffer (20mM Tris, 250 mM sucrose, 1 mM EDTA, 2 mM PMSF, 2 tablets of protease inhibitor cocktail, pH 7.4) was used instead of RIPA lysis buffer in order to measure the amount of GLUT4 in total cellular membrane protein [12,13]. Protein content was determined by the BCA method as described above. The proteins of each sample were boiled for 5 minutes, separated in 10% SDS-PAGE and transferred to a nitrocellulose membrane. Membranes were blocked with 5% non-fat milk in TBST (200 mM Tris base, 1.37 mM NaCl and 0.1% Tween-20) pH 7.6 for 1 hour and then incubated with primary antibody overnight at 4°C. The primary antibodies were used as following: GLUT4 (1F8), Cell Signaling, 1:1000; Phospho-Akt (Ser473), Cell Signaling, 1:500; Phospho-AMPKα (Thr172), Cell Signaling, 1:500; Akt, Cell Signaling, 1:1000; AMPKα, Cell Signaling, 1:1000 or β-actin, Cell Signaling, 1:1000. After washing, the membranes were then incubated for 1 hour at room temperature with horseradish-peroxidase-conjugated secondary antibodies (anti-rabbit, Jackson, 1:20000 or anti-mouse IgG, Cell Signaling, 1:2000). The bands were detected by enhanced chemiluminescence (ECL) method and were quantified using NIH Image J 1.45s software (National Institutes of Health, Bethesda, MD, USA). All experiments were conducted on 3 different cell preparations.

### Statistical analysis

Results are presented as mean ± SEM of 3 independent experiments. Statistical calculations were performed with Prism 5 (GraphPad Software Inc., San Diego, CA, USA). Differences between EE (or HWE) and vehicle controls were analyzed by one-way analysis of variance (ANOVA) and post hoc Dunnett's test. And differences between EE and HWE were analyzed by two-way analysis of variance (ANOVA) and post hoc Bonferroni’s test.

## Results

### Stimulation of glucose uptake in myotubes by different plant extracts

**Fig 1:**
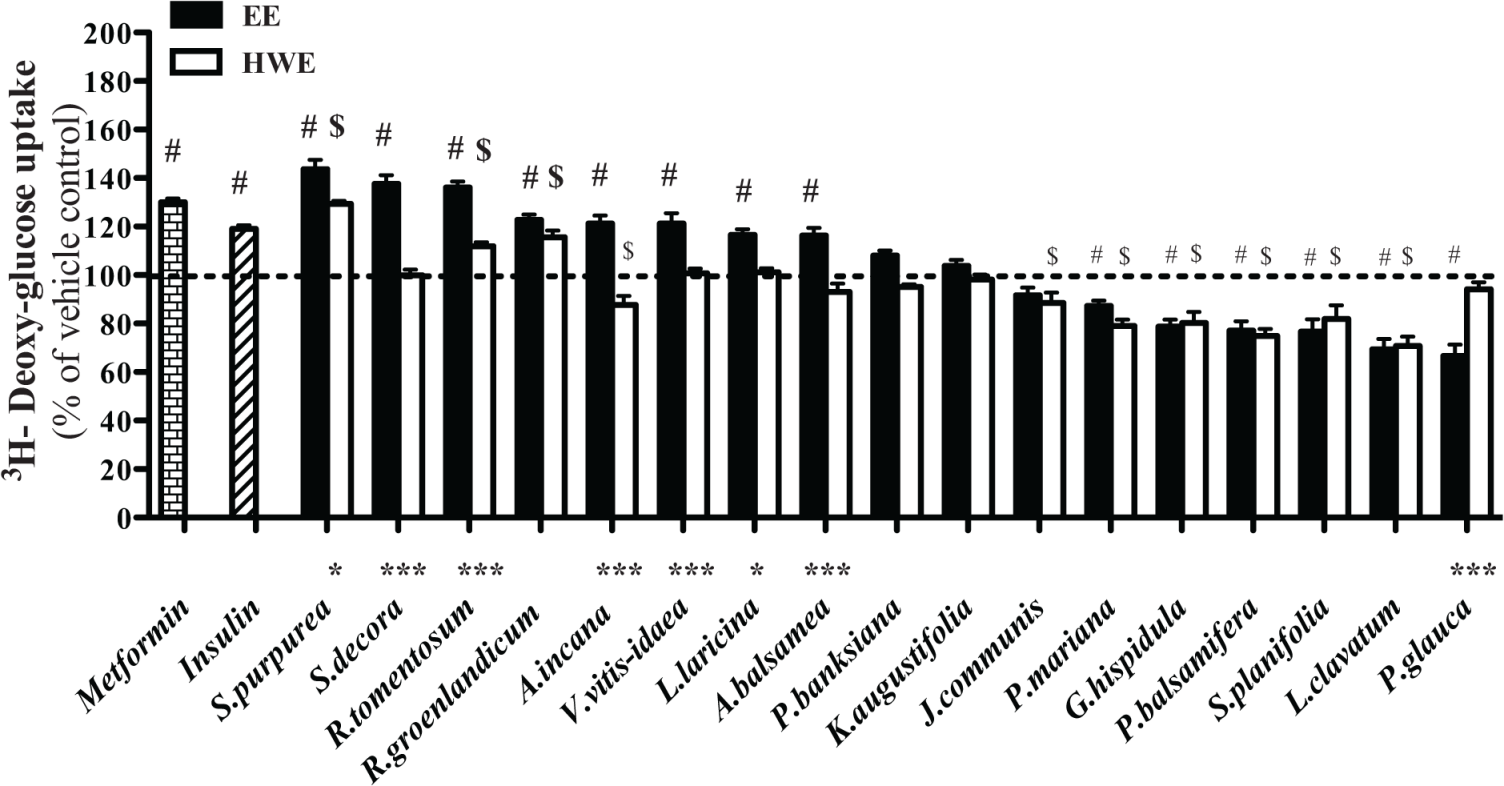
Effects of extracts on muscle glucose transport. C2C12 skeletal muscle cells were treated with either 0.1% DMSO (vehicle), Metformin (400 μM), EE and HWE (at concentrations described in Table 1) for 18 hours, or with insulin (100 nM) for 30 min. Results represent means ± SE for 3 separate experiments, normalized to the vehicle-treated condition. # Denotes EE samples significantly different from vehicle control (*p* < 0.05), one-way ANOVA and post hoc Dunnett's test. $ Denotes HWE samples significantly different from vehicle control (p < 0.05), one-way ANOVA and post hoc Dunnett's test. * (*p* < 0.05), **(*p* < 0.01) and *** (*p* < 0.001) denote significant differences between respective EE and HWE counterparts, two-way ANOVA.

Plant extracts were tested for enhancing glucose transport properties in an insulin-responsive and GLUT4-containing cell line, namely, C2C12 myoblasts [14,15]. Metformin (400 μM, 18 hours) and insulin (100 nM, 30 minutes), applied as positive controls, significantly stimulated glucose transport to 130% and 119% of DMSO vehicle values, respectively. Eight out of the 17 EE of plants (namely, *S*. *purpurea*, *S*. *decora*, *R*. *groenlandicum*, *R*. *tomentosum*, *A*. *incana*, *V*. *vitis-idaea*, *L*. *laricina* and *A*. *balsamea*) enhanced glucose uptake in C2C12 cells after 18 hours incubation. Among these, *S*. *purpurea* showed the strongest potential (144% compared with vehicle control) (Fig 1). In the case of HWE, only 3 species (namely, *R*. *groenlandicum*, *S*. *purpurea*, and *R*. *tomentosum*) also elicited significant increases in glucose uptake. Only the HWE of *R*. *groenlandicum* exhibited an effect as strong as its EE. The HWE of *S*. *purpurea* and *R*. *tomentosum* exhibited a decreased effectiveness compared to the stimulation of their EE. For other species (such as *A*. *incana*), their HWE completely lost their effect in C2C12 cells as compared to their EE counterparts.

### Inhibition of hepatic glucose production and G6Pase activity of extracts

**Fig 2:**
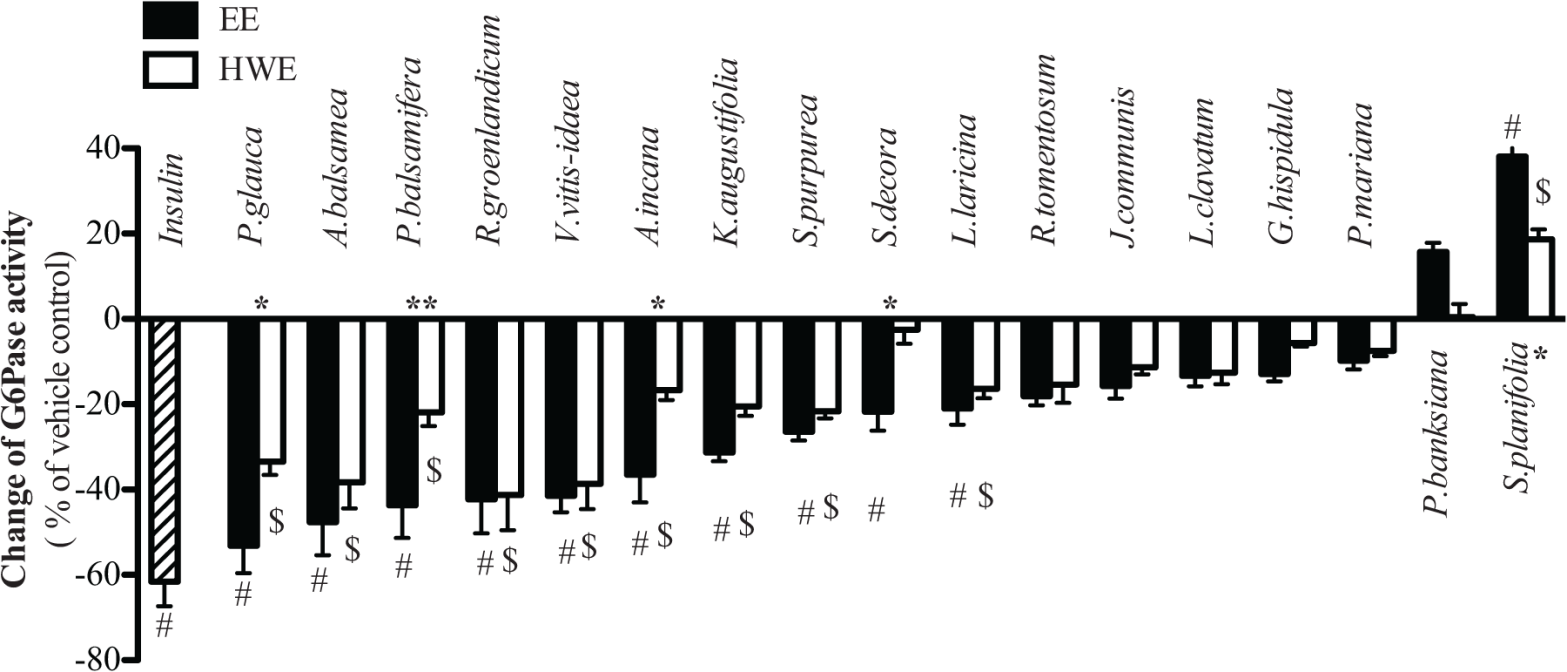
Effects of extracts on hepatic Glucose-6-phosphatase activity. H4IIE rat hepatoma cells were treated for 18 hours with vehicle control, or insulin (100 nM), EE or HWE plants extracts (at concentrations as described in Table 1). Results represent means ± SE for 3 separate experiments, normalized to the vehicle-treated condition. # Denotes EE samples significantly different from vehicle control (p < 0.05), one-way ANOVA and post hoc Dunnett's test. $ Denotes HWE samples significantly different from vehicle control (p < 0.05), one-way ANOVA and post hoc Dunnett's test. * (*p* < 0.05), **(*p* < 0.01) and *** (*p* < 0.001) denote significant differences between EE and HWE counterpart, Two-way ANOVA.

Insulin 100 nM, applied as a positive control to H4IIE liver cells, inhibited G6Pase activity by 62% compared with vehicle control (Fig 2). When the Cree plant extracts were tested, a statistically significant reduction in G6Pase activity (varying from -54% to -17% compared with vehicle control) was observed for both the EE and HWE of 9 species (*P*. *glauca*, *A*. *balsamea*, *P*. *balsamifera*, *R*. *groenlandicum*, *K*. *angustifolia*, *S*. *purpurea*, *A*. *incana*, *L*. *laricina*, and *V*. *vitis-idaea*), as well as by the EE of one species (*S*. *decora*). Hence, the inhibitory potential of most active species was maintained at a near equivalent level when HWE was used instead of EE. In three cases (*P*. *glauca*, *P*. *balsamifera* and *A*. *incana*), HWE biological activity on G6Pase was significantly lower (-34%, -22%, -17% respectively) when compared with the respective EE counterpart (-54%, -44%, -37% respectively), whereas for *S*. *decora* the inhibitory effect of its EE (-22%) was completely lost with its HWE.

### Effects of selected CEI plant extracts on expression of GLUT4 and phosphorylation of AMPK and Akt in C2C12 myotubes

Five plants, namely *R*. *groenlandicum*, *K*. *angustifolia*, *S*. *purpurea*, *A*. *incana* and *P*. *balsamifera* were selected for further in-depth pharmacological analysis. The selection process sought to capture representative situations encountered, namely, species with or without biological activity in our bioassays, as well as species with varying or stable biological activities between their EE and HWE.

**Fig 3:**
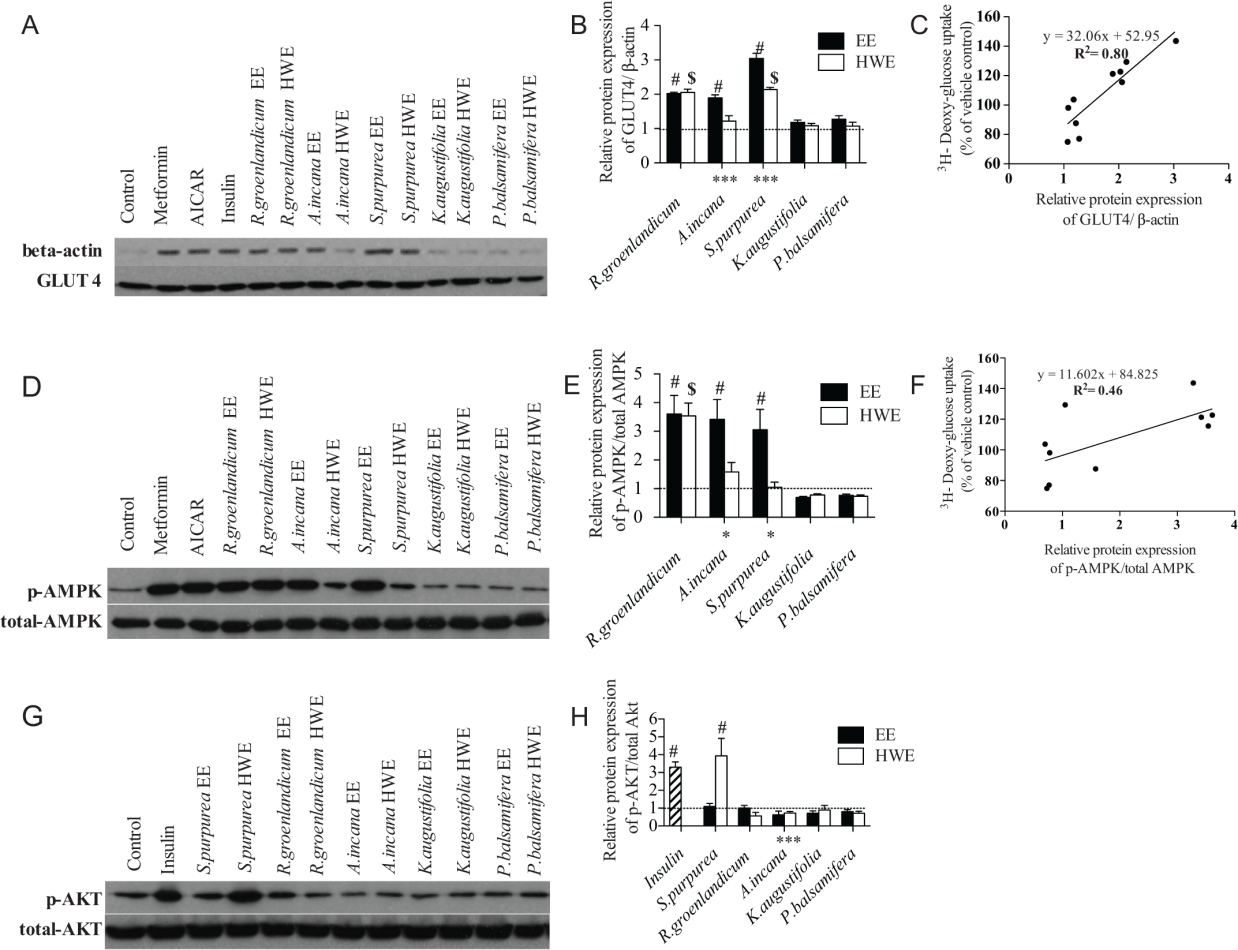
Effect of selected extracts on expression of GLUT4, Insulin and AMPK pathway in C2C12 cells. Cells were differentiated and treated for 18 hours with vehicle or with EE and HWE of the 5 selected plants. Metformin (400 μM, 18 hours), AICAR (2 mM, 2 hours) or insulin (100 nM, 30 min) was applied as positive controls for the AMPK or insulin pathways, respectively. GLUT4 (3A), phosphorylation of AMPK (3D), phosphorylation of Akt (3G) were measured by western blot and results (3B, 3E, 3H) were expressed as means ± SE for 3 separate experiments, normalized to the vehicle-treated condition. # Denotes EE samples significantly different from vehicle control (p < 0.05), one-way ANOVA and post hoc Dunnett's test. $ Denotes HWE samples significantly different from vehicle control (*p* < 0.05), one-way ANOVA and post hoc Dunnett's test. * (*p* < 0.05), **(*p* < 0.01) and *** (*p* < 0.001) denote significant differences between EE and HWE counterpart, two-way ANOVA. Correlation results (3C, 3F) were analyzed by linear regression and the equations were y = 32.06x + 52.95 (R = 0.80, *p* < 0.05), y = 11.602x + 84.825 (R = 0.46, *p* < 0.05), respectively.

To begin understanding the mechanisms underlying the observed effect of extracts on muscle cell glucose regulation, we evaluated the total membrane protein expression of GLUT4, the insulin-dependent kinase, Akt, and the insulin-independent AMPK pathway (the latter two representing main signaling pathways regulating glucose uptake in this cell line). Insulin served as a positive control for the first pathway, whereas metformin and AICAR were applied as activators of AMPK signaling. At the end of treatments in C2C12 cells, there was a clear increase in the expression of GLUT4 with all three positive controls as compared with vehicle (2.8-fold, 2.7-fold and 2.3-fold increases for insulin, metformin and AICAR respectively, Fig 3A). *R*. *groenlandicum* represents a species whose HWE and EE had comparable effects on glucose uptake. Both its extracts also stimulated the expression of GLUT4 (2.0-fold for both HWE and EE, Fig 3A and 3B), as well as AMPK phosphorylation (3.6-fold and 3.5-fold respectively, Fig 3D and 3E) in a similar manner, whereas Akt was not affected by either extract. In contrast, *A*. *incana* represents a species whose HWE completely lost its potentiating effect on GU as compared with its EE. Consistent with this, *A*. *incana* HWE was without effect on C2C12 GLUT4 content and AMPK phosphorylation whereas its EE increased both parameters by 1.9-fold (Fig 3A and 3B) and 3.4-fold (Fig 3D and 3E), respectively. In fact, linear regression analysis clearly demonstrated that effects of selected species on glucose uptake were tightly correlated with their corresponding impact on C2C12 GLUT4 content (R^2^ = 0.80, p < 0.05; Fig 3C). A similar, albeit less tight, correlation was also observed between a given plant extract’s ability to increase glucose uptake and to enhance AMPK phosphorylation (R^2^ = 0.46, p < 0.05, Fig 3F).

On the other hand, *S*. *purpurea* was a species that yielded interesting observations. As shown in Fig 1, its HWE significantly stimulated GU, although at lower levels than its EE. When underlying mechanisms were probed, GLUT4 expression results were consistent with GU results (EE greater than HWE). However, *S*. *purpurea* HWE was the only extract found to significantly enhance Akt phosphorylation (3.9-fold increase, p < 0.05; Fig 3G and 3H), yet was without effect on AMPK phosphorylation (Fig 3D and 3E). Conversely, the plant’s EE stimulated AMPK phosphorylation (3.1-fold, p < 0.05; Fig 3D and 3E) without impacting Akt phosphorylation.

### Effects of the selected extracts on phosphorylation of AMPK and Akt by in H4IIE cells

We used the same positive controls and probed the same molecular parameters (except GLUT4 that is absent in hepatocytes) to determine the latters’ implication in the selected plant extracts’ actions on hepatic glucose production.

**Fig 4:**
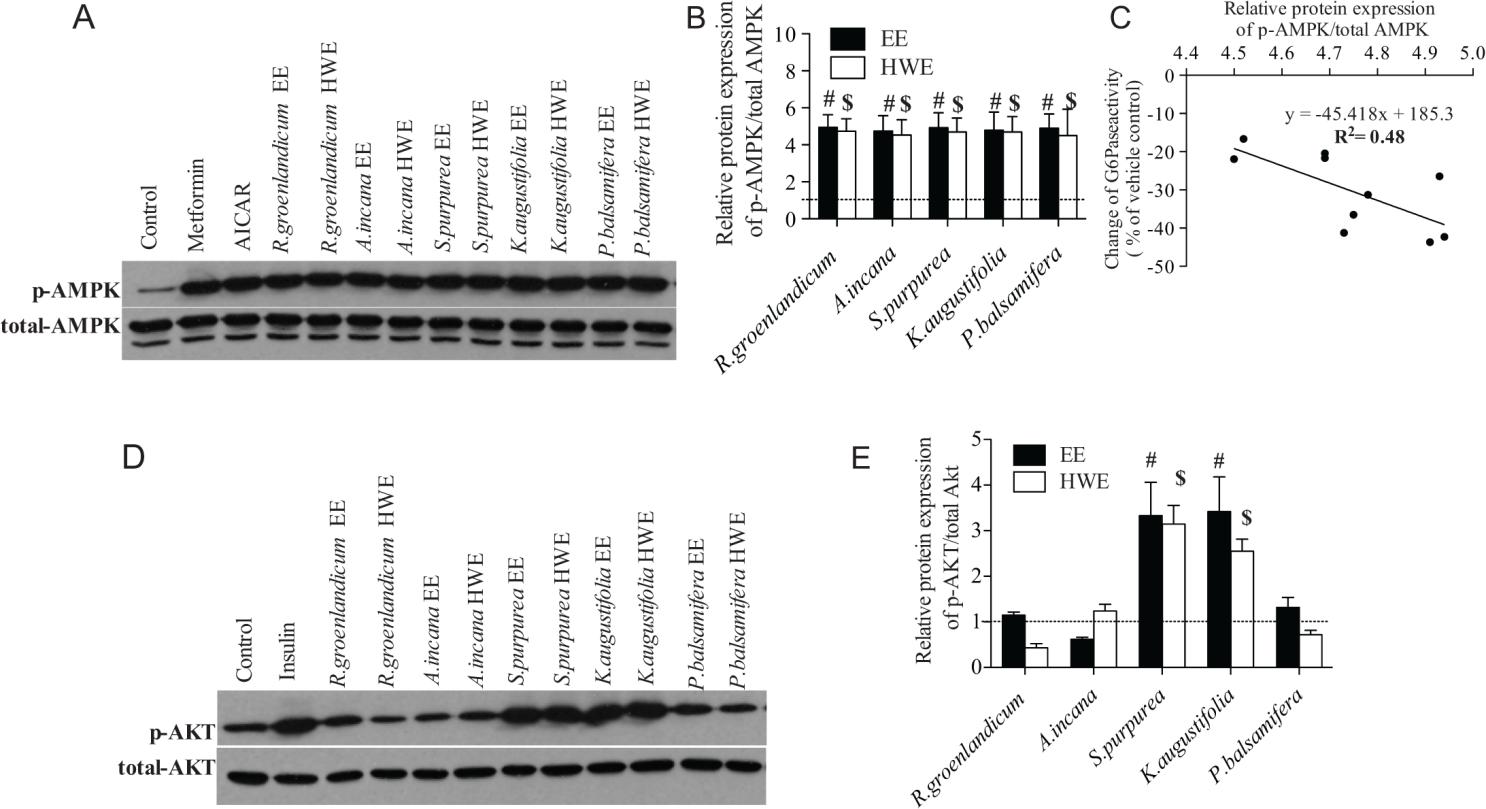
Effect of selected extracts on the modulation of insulin and AMPK pathway in H4IIE cells. The cells were treated for 18h with vehicle control, insulin (100 nM), EE or HWE plants extracts. Metformin (400 μM, 18 hours), AICAR (2 mM, 2 hours) or insulin (100 nM, 18 hours) was used as positive controls for AMPK or insulin pathways, respectively. Phosphorylation of AMPK (4A) and of Akt (4D) was measured by western blot and results (4B, 4E) expressed as means ± SE for 3 separate experiments, normalized to the vehicle-treated condition. # Denotes EE samples significantly different from vehicle control (p < 0.05), one-way ANOVA and post hoc Dunnett's test. $ Denotes HWE samples significantly different from vehicle control (p < 0.05), one-way ANOVA and post hoc Dunnett's test. Correlation results (4C) were analyzed by linear regression and the equation was y = -45.418x + 285.3 (R = 0.48, *p* < 0.05).

In H4IIE hepatocytes, the EE and HWE of all selected plant species increased AMPK phosphorylation with similar intensity (around 4-fold compared with vehicle, p < 0.05; Fig 4A and 4B). Despite this, for a given plant, linear regression analysis uncovered a statistically significant correlation between the stimulation of AMPK and the inhibition of G6Pase (R^2^ = 0.48, p < 0.05, Fig 4C). In contrast, only *S*. *purpurea* and *K*. *angustifolia* were found capable of stimulating Akt phosphorylation (by 2-3-fold, p < 0.05; Fig 4D and 4E); this effect being comparable in EE and HWE crude preparations.

### Metabolomics analysis

**Fig 5:**
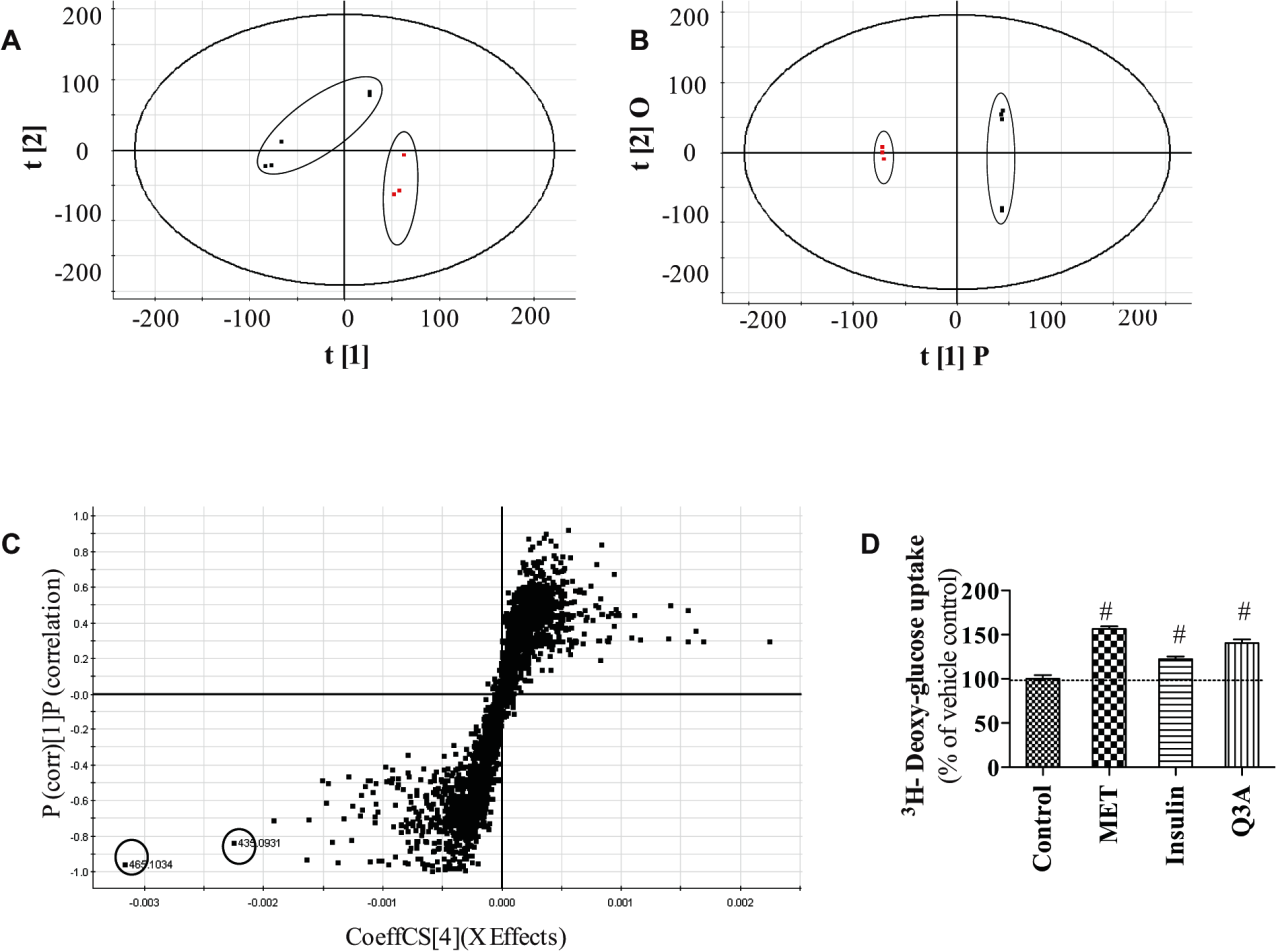
Metabolites analysis of selected hot water extracts based on stimulating glucose uptake activity. PCA scores (5A), OPLS-DA scores (5B) S-plot (5C) of UPLC-QTOF metabolome of HWE of Cree plants. Those stimulating glucose transport (*R*. *gromenlandicum*, *R*. *tomentosum*, and *S*. *purpurea*) grouped seperately from inactive ones (*K*. *angusfolia* was found to be an outlier and was excluded from the process). The 95% confidence interval for each group is given. In the S-plot, the metabolome of the active plants was compared with the inactive plants to identify discriminant biomarkers with *K*. *angustifolia* excluded. In Fig 5D, quercetin 3-O-α-L-arabinopyranoside (Q3A, 50 μM) stimulated GU in C2C12 cells, 140% compared with vehicle control.

Using the optimal UPLC–QTOF conditions described above, representative fingerprints for all HWE and EE of the 17 plants were obtained. Overall, more than 4000 metabolites were detected from these extracts by UPLC-QTOF. This generated a matrix of data comprising qualitative (relating to phytochemical compounds, notably retention time, elemental composition and accurate mass) and relative quantitative (signal intensities) components. Principal component analysis (PCA) was then used to assess the relationships between phytochemical metabolites (profiles) of the different plant extracts (Fig 5). Using an unbiased PCA approach including HWE and EE, as well as all species regardless of their biological phenotype (e.g. active versus inactive for a given bioassay), there were no clear groupings. We then carried out more specific PCA groupings guided by the biological activity found in our bioassays (presence or absence of effects on muscle glucose uptake or hepatic G6Pase activity) and for HWE and EE samples separately.

We first examined EE preparations and compared species with a stimulating action on GU with inactive ones using PCA, but no discriminating characteristics were observed. Similarly, when EE or HWE samples inhibiting G6Pase were analyzed against corresponding inactive species, the PCA process did not show any clustering.

In contrast, when the HWE preparations of the 17 plants were analyzed by PCA a clear discrimination of species was obtained (Fig 5A and 5B). This led to two groupings, namely extracts with and without a capacity to stimulate glucose uptake in C2C12 cells. Indeed, plants stimulating glucose uptake, namely *R*. *groenlandicum*, *S*. *purpurea* and *R*. *tomentosum*, clustered together. The 95% confidence interval for this grouping is shown. Similarly, inactive species (*A*. *balsamea*, *L*. *laricina*, *S*.*decora*, *P*. *glauca*, *P*. *banksiana*, and *V*. *vitis-idaea*) were found to group together within the 95% confidence shown. *K*. *augusfolia* was an outlier and thus excluded from the analysis.

A so-called S-plot was then generated to determine plant metabolites that could significantly contribute to discriminating the active group of species from the inactive one. These metabolites usually lie in the top or bottom p_corr_ values and the extremities of the x-axis in the S-plot, and are characterized by sufficiently important statistical differences to render them potential biomarkers for the biological activity studied. As shown in Fig 5B and 5C, two markers were identified as the potential biomarkers from S-plots.

The identify of two biomarkers was based on UPLC-QTOF analysis, performed in positive ion mode using MSe settings which simultaneously produced low (6V) and high (20–50V) fragmentation spectra for each peak above the noise threshold. Molecular ion and molecular formula was searched in ChemSpider (http://www.chemspider.com), Metlin (http://metlin.scripps.edu) while mass fragment was used for further confirmation of identity. The identity of the markers was finalized by multiple reaction monitoring on a 3200 QTRAP using authentic standards. The two biomarkers were identified as Quercetin 3-O-α-L-arabinopyranoside (with retention time 3.09 min, monoisotopic mass of [M+H]^+^ 435.0931) and Quercetin-3-O-galactoside (with retention time 2.83 min, monoisotopic mass of [M+H]^+^ 465.1034), respectively.

Quercetin-3-O-galactoside was previously isolated by our group using bioassay-guided fractionation and showed a significantly stimulating effect on glucose transport (16). Here our results showed that quercetin 3-O-α-L-arabinopyranoside also stimulated GU in C2C12 cells (140% compared with vehicle control, Fig 5D).

## Discussion

Through ethnobotanical surveys, our group identified 17 medicinal plants as candidate antidiabetic species. [2, 9] We found that several CEI plants’ ethanol extracts strongly potentiated glucose transport in C2C12 cells, whereas another group of species significantly inhibited G6Pase in H4IIE liver cells, indicating a potential to increase peripheral glucose disposition and to suppress hepatic glucose production, respectively. [5,7] In the present study, we used the same bioassays to evaluate the antidiabetic potential of a more traditional HWE preparation and compare it with the standard phytochemical ethanol extraction procedure. We also sought to begin assessing the potential underlying molecular mechanisms in this in vitro system.

Generally in line with previous observations by our group [2,4], our results showed that several EE of CEI plants enhanced glucose transport in C2C12 differentiated myoblasts. The present studies demonstrated that three species (both *Rhododendron* spp. and *S*. *purpurea*) completely or partially maintained their capacity to stimulate such glucose transport when more traditional HWE preparations were used. Five other species completely lost this biological activity in similar conditions. All inactive species remained inactive when HWE was used instead of EE. These results could be interpreted to mean that more traditional preparations are not as “good” as the classical ethanol extraction procedure used in phytochemistry. However, a number of considerations can argue differently. Firstly, EE and HWE extracts were tested at identical mg/mL concentrations of the crude extracts for purposes of proper pharmacological comparison. Since ethanol is known to be a better solvent than water to extract several bioactive plant molecules (notably of the large class of phenolic compounds), our observations could simply reflect the fact that certain bio-actives were more concentrated in EE and hence were able to enhance biological activity. Future studies should use wide ranges of extract concentrations to better compare the pharmacological potential of EE and HWE plant preparations. Secondly, the laboratory method used to prepare the HWE mimicked procedures used by traditional CEI healers and was indeed adjusted after discussions with them. However, traditional healers may use lake water instead of distilled water and different heating conditions than were used in the laboratory. Hence, future studies should compare laboratory HWE with true traditional preparations of CEI healers. Nonetheless, the present results confirmed the soundness of our current and previous studies to assess the biological activity of putative antidiabetic CEI plant species. Indeed, we found that the capacity of both EE and HWE preparations of five representative species to increase glucose transport strongly correlated with their extracts’ ability to increase GLUT4 protein content in C2C12 cells. Hence, the potential of the various extracts to enhance peripheral glucose disposition is related to their action on the major glucose transport protein of skeletal muscle. However, future studies will be necessary to confirm that the plants increased GLUT4 translocation. As importantly, a significant, albeit somewhat weaker, correlation was observed between increased glucose transport and AMPK phosphorylation for both EE and HWE preparations tested. This is in full agreement with what was described in our previous study on a subset of eight CEI plants [16]. Indeed, we had shown that glucose uptake was increased by several species though the AMPK pathway rather than the insulin-dependent pathway involving Akt. The insulin-independent AMPK signaling pathway is also the one through which the common oral hypoglycemic drug, metformin, which stimulate glucose transport in skeletal muscle, inhibits hepatic glucose production and improves insulin sensitivity [17,18].

On the other hand, we made a very interesting observation concerning the species *Sarracenia purpurea* L., (or namely the purple pitcher plant), which strongly stimulates C2C12 glucose uptake. We found that the EE of *S*. *purpurea* triggered significant AMPK phosphorylation (activation), whereas its HWE counterpart was the only one of all species tested to activate Akt phosphorylation, which is related to the insulin pathway. In parallel, the capacity of *S*. *purpurea* HWE to stimulate glucose transport was lower than the corresponding EE. In previous studies, we had tested the effect of the same plant’s EE in the same bioassay and in the presence or absence of physiological and pharmacological concentrations of insulin. [5] We found that EE of *S*. *purpurea* had a tendency to increase the glucose uptake induced by insulin, consistent with an action on the “insulin-sensitizing” AMPK pathway. We also isolated several bioactive compounds from *S*. *purpurea* EE using C2C12 cells to guide a fractionation process and some of these compounds were also shown to activate AMPK. [11, 16] The present studies thus suggest that the HWE of *S*. *purpurea* may contain other compounds that activate, instead, the insulin-dependent pathway involving Akt. Future studies using a similar bioassay-guided fractionation approach with HWE may clarify this interpretation.

The second assay related to glucose homeostasis that we have used is based on the inhibition of G6Pase in H4IIE liver cells as an indicator of an antidiabetic potential to reduce hepatic glucose production. Our results clearly showed that several species had a significant inhibitory effect on G6Pase. Unlike the C2C12 bioassay, however, the inhibitory action of most HWE was similar to that of their EE counterparts, only three species showing lower activities and only one losing it completely with HWE. Likewise, all five of the selected subset of representative species enhanced AMPK phosphorylation and this was equivalent between the plants’ respective EE and HWE. This is consistent with the reported effects of metformin and many natural products that inhibit hepatic glucose production in liver [19–21]. In addition, two plants extracts (*S*. *purpurea* and *K*. *angustifolia*) also activated insulin pathways by inducing Akt phosphorylation; this being true for both their respective EE and HWE. The differences in results comparing EE and HWE in C2C12 differentiated myoblasts and H4IIE hepatocytes demonstrate that in some cases, bioactive components (and combinations thereof) of CEI plants can be extracted with similar efficiency by the two extraction methods used.

*R*. *groenlandicum*, (known as *Ledum groenlandicum* or Labrador tea), is a good case for this point. It is a flowering plant in the subsection Ledum of the large genus *Rhododendron* in the *Ericaceae* family. Leaves and other parts have shown antioxidant and anti-inflammatory [22] activity, as well as benefits against cancer [23] asthma [24], rheumatism [25] and diseases of the kidney [24]. In the present studies, *R*. *groenlandicum* was the only specie whose EE and HWE preparations were found to be active in both assays and to exert their antidiabetic effect through similar molecular pathways.

*R*. *groenlandicum* was also one of three species stimulating glucose transport (alongside *S*. *purpurea* and *R*. *tomentosum*) whose HWE clustered in a way that was clearly separated from inactive species, when photochemical profiling and PCA process were applied to our data. Most importantly, analysis using the discriminant analysis uncovered significant differences in the chemical profiles of these plants. Notably, two biomarkers were identified with a strong potential to be responsible for the biological activity of the active plants, due to their highest contribution to this separation of clustering. One of these, quercetin-3-O-galactoside, was previously identified by our team and reported to be one of the active principles capable of enhancing glucose transport in C2C12 through the activation of AMPK. [11, 16] On the other hand, the second putative antidiabetic compound, identified in the present report as quercetin 3-O-α-L-arabinopyranoside, has not been isolated in prior bioassay-guided fractionation studies by our group or associated with this biological activity by others. However, because of the labor intensive and tedious characteristics of the bioassay-guided fractionation approach, only the most active subfractions were selected for the identification of active principles. Hence, other active metabolites may exist in other fractions of the active species and the active quercetin 3-O-α-L-arabinopyranoside might thus contribute to the antidiabetic potential of some CEI plant species.

## Conclusion

In summary, we evaluated and compared the potentials of the EE and HWE of 17 plants to modulate glucose homeostasis in muscle and liver cells, two major targets of insulin action. We also assessed the possible molecular mechanisms responsible for such activities in vitro. We confirmed that the different species, target tissues or cells (muscle or liver), as well as extraction methods (EE or HWE), are all significant determinants of the biological activity of Cree medicinal plants on glucose metabolism. Applying principal component analyses to mass spectrometric data obtained by UPLC-QTOF enabled us to segregate the metabolomes of active plant species from the inactive ones. This powerful approach offers an interesting avenue to identify or confirm potential bioactive plant metabolites. To our knowledge, this is the first report where metabolomics analysis has confirmed results obtained through bioassay-guided fractionation. Our work and its future applications can help to develop novel as well as culturally relevant plant-based therapeutic approaches against insulin resistance that target muscle and liver cells directly. Finally, our studies can serve as quality control tools to foster reliable and effective plant-based treatments, using the information relative to the content of active principles as well as that pertaining to biological activity.
